# A pragmatic randomized trial of a primary care antimicrobial stewardship intervention in Ontario, Canada

**DOI:** 10.1186/s12875-021-01536-3

**Published:** 2021-09-15

**Authors:** Warren McIsaac, Sahana Kukan, Ella Huszti, Leah Szadkowski, Braden O’Neill, Sophia Virani, Noah Ivers, Rosemarie Lall, Navsheer Toor, Mruna Shah, Ruby Alvi, Aashka Bhatt, Yoshiko Nakamachi, Andrew M. Morris

**Affiliations:** 1 Department of Family and Community Medicine, University of Toronto, Toronto, Canada; 2 Ray D. Wolfe Department of Family Medicine, Mount Sinai Hospital, Sinai Health, 60 Murray St, Toronto, ON M5T 3L9 Canada; 3 Biostatistics Research Unit, University Health Network, Toronto, Canada; 4 Department of Family and Community Medicine, St. Michael’s Hospital, Toronto, Canada; 5 Li Ka Shing Knowledge Institute, St. Michael’s Hospital, Toronto, Canada; 6 Department of Family Medicine, and Women’s College Research Institute, Women’s College Hospital, Toronto, Canada; 7 Institute of Health Policy, Management and Evaluation, University of Toronto, Toronto, Canada; 8 Platinum Medical, Scarborough Health Network Teaching Unit, Toronto, Canada; 9 Southlake Academic Family Health Team, Southlake Regional Health Centre, Newmarket, Toronto, Ontario Canada; 10 West Durham Family Health Team, Pickering, Toronto, Ontario Canada; 11 Summerville Family Health Team, Mississauga, Ontario Canada; 12 Antimicrobial Stewardship Program, University Health Network, Toronto, Canada; 13 Department of Medicine, Division of Infectious Diseases, Sinai Health, University Health Network, and University of Toronto, Toronto, Canada

**Keywords:** Antimicrobial stewardship, Primary care, Controlled trial

## Abstract

**Background:**

More than 90% of antibiotics are prescribed in primary care, but 50% may be unnecessary. Reducing unnecessary antibiotic overuse is needed to limit antimicrobial resistance. We conducted a pragmatic trial of a primary care provider-focused antimicrobial stewardship intervention to reduce antibiotic prescriptions in primary care.

**Methods:**

Primary care practitioners from six primary care clinics in Toronto, Ontario were assigned to intervention or control groups to evaluate the effectiveness of a multi-faceted intervention for reducing antibiotic prescriptions to adults with respiratory and urinary tract infections. The intervention included provider education, clinical decision aids, and audit and feedback of antibiotic prescribing. The primary outcome was total antibiotic prescriptions for these infections. Secondary outcomes were delayed prescriptions, prescriptions longer than 7 days, recommended antibiotic use, and outcomes for individual infections. Generalized estimating equations were used to estimate treatment effects, adjusting for clustering by clinic and baseline differences.

**Results:**

There were 1682 encounters involving 54 primary care providers from January until May 31, 2019. In intervention clinics, the odds of any antibiotic prescription was reduced 22% (adjusted Odds Ratio (OR) = 0.78; 95% Confidence Interval (CI) = 0.64.0.96). The odds that a delay in filling a prescription was recommended was increased (adjusted OR=2.29; 95% CI=1.37, 3.83), while prescription durations greater than 7 days were reduced (adjusted OR=0.24; 95% CI=0.13, 0.43). Recommended antibiotic use was similar in control (85.4%) and intervention clinics (91.8%, p=0.37).

**Conclusions:**

A community-based, primary care provider-focused antimicrobial stewardship intervention was associated with a reduced likelihood of antibiotic prescriptions for respiratory and urinary infections, an increase in delayed prescriptions, and reduced prescription durations.

**Trial registration:**

clinicaltrials.gov (NCT03517215).

**Supplementary Information:**

The online version contains supplementary material available at 10.1186/s12875-021-01536-3.

## Background

The need to more appropriately use antibiotics in order to limit antibiotic resistance has been widely endorsed [1, 2]. However, progress towards this goal has been slow. In Canada for example, antibiotic utilization has changed little, with 666 community antibiotic prescriptions dispensed per 1,000 persons in 2012 and 658 prescriptions in 2017 [3]. The recognition that 90% of antibiotic use occurs in the community has focused attention on primary care prescribers [4]. A 2019 U.S. study found 59% of outpatient antibiotic prescriptions are likely not needed [5]. Reducing this volume of unnecessary antibiotic use may be critical to limit antibiotic resistance. To achieve this, effective community antimicrobial stewardship (AMS) approaches are needed.

Community-based AMS programs have been tested in Scotland [6], Spain [7], the United States [8], the Netherlands [9], the United Kingdom [10], and Canada [11]. Most involved education and prescribing guidelines combined with audit-and-feedback [8–11]. Some were limited to either specific organisms [7], short durations [8], respiratory infections [9], effects were small [10], or evaluations lacked control groups, limiting conclusions about effectiveness [11, 12]. Some providers report a lack of confidence in AMS approaches, raising questions about whether community prescribers would implement programs, even where effective [13, 14]. To ensure AMS is relevant to community prescribers, programs may need to ensure they are addressing the prescribing challenges that community providers feel are most important.

One factor may be the clinical uncertainty community providers report in trying to distinguish viral from bacterial infections, owing to their similar clinical presentations [15]. There are also few point-of-care tests to aid in identifying bacterial presentations that might benefit from antibiotics. We previously developed a multi-faceted primary care provider-focused antimicrobial stewardship intervention that addressed clinical uncertainty through use of clinical decision aids, delayed prescriptions and safety netting advice [16]. A pilot evaluation identified competing clinical commitments, perceived pressure to prescribe antibiotics, and a lack of resources for stewardship activities as additional barriers for community clinicians [16]. The objective of this study was to assess the effectiveness of a community-based primary care provider-focused antimicrobial stewardship intervention addressing clinical uncertainty and other barriers on antibiotic prescription practices of primary care providers for common community infections.

## Methods

### Study setting and design

The University of Toronto Practice-based Research Network (UTOPIAN) consists of 14 family medicine clinics in south central Ontario affiliated with the Department of Family and Community Medicine of the University of Toronto. Two clinics had participated in a pilot study of the intervention and were excluded [16]. Of the remaining 12 clinics invited to participate, family physicians and nurse practitioners from six clinics agreed to participate in a pragmatic controlled trial of a primary care provider-focused stewardship intervention. The intervention was delivered between September 2018 and December 2018, followed by a 5 month evaluation period from January 1^st^ 2019 until May 31^st^ 2019. Baseline prescribing rates for each clinic were calculated from prescribing data for the previous winter. Ethics approval was obtained from research ethics boards of the University of Toronto, Mount Sinai Hospital, Women’s College Hospital and North York General Hospital. The study was registered with clinicaltrials.gov on 08/05/2018 (registration number NCT03517215).

Randomization by minimization was conducted due to the small number of clinics [17]. Using prescribing data collected for the winter before the intervention, prescribing rates, number of providers and the presence of trainees were selected as minimizing factors. Clinics were assigned a number (by SV) and randomly assigned (by WM) without awareness of clinic identities. However, delays in securing participation from all clinics led to five being initially randomized, and a sixth clinic was later allocated to balance provider numbers. As a result, imbalances in factors associated with antibiotic use at baseline persisted. Statistical methods were therefore utilized to adjust for these factors in estimating the effect of the intervention.

### Intervention

The intervention was a multi-faceted program of clinician education, clinical decision aids for prescribing decisions, patient information leaflets, audit and feedback of clinic prescription practices, local clinic support, and incentives. An initial one hour on-site education session was delivered by study staff at each clinic regarding antimicrobial resistance, stewardship, and interventions for reducing antibiotic prescribing. The clinic’s prescribing practices the previous winter were reviewed, and providers set prescribing goals. Providers were then sent electronic modules to complete over four months explaining the use of the decision aids for a given condition and optimal prescription practices. Additional on-site sessions were held during the winter to review antibiotic utilization and revise prescribing goals.

The modules addressed five infections: acute sinusitis, acute uncomplicated upper respiratory infections (URI), sore throat presentations (pharyngitis, tonsillitis), acute bronchitis and acute uncomplicated cystitis. These conditions account for approximately 50% of community antibiotic prescriptions in Canada [18]. Modules took approximately 15 minutes to complete and were sent each month by email to intervention clinics.

Module topics included prescribing issues for each infection and a 1-page clinical prescribing decision aid. The aid addressed criteria for diagnosis, indications for antibiotics, recommended first line antibiotic choices, treatment durations, and ‘red flags’ for serious presentations. Validated clinical decision rules were incorporated into prescribing aides where available [19, 20]. Modules also included ‘communication’ scripts to engage patients in prescribing decisions [21], patient handouts, delayed prescription options, [22] and advice to give patients about when to seek medical care where antibiotics were not prescribed (‘safety-netting’) [23].

Clinicians at intervention sites received $200 compensation, pro-rated for the number of completed modules and education sessions. Continuing medical education credits and a free antibiotic prescribing formulary [24] were also provided. Control arm participants provided usual care but received the free antibiotic formulary upon trial completion.

### Data collection

Data from eligible visit encounters were abstracted from electronic medical record (EMR) systems at each clinic. Eligible visits were defined as those involving adults 18 years of age or older, seen by a consenting physician between January 1^st^ 2018 and February 28^th^ 2018 (baseline period) or between January 1^st^ 2019 and May 31^st^ 2019 (evaluation period), and with an eligible ICD-9 diagnosis code (International Classification of Diseases, Ninth Revision (ICD 9) identified from billing records. Urinary infections involving males or pregnant females were excluded, as were follow-up visits of previously treated infections.

To identify most eligible visits, a number of ICD-9 billing codes were selected. These included 460 (URI), 462 (pharyngitis), 463 (tonsillitis), 461(acute sinusitis), 466 (acute bronchitis), and (595) acute uncomplicated cystitis. In addition, visits coded 464 (laryngitis), 599 (other urinary-eg. hematuria, incontinence), 486 (pneumonia), and 487(influenza) were included to ensure other respiratory and urinary symptom visits had not been mislabeled. This was assessed by comparing a provider’s written diagnosis and the billing code. Two independent raters reviewed all visits using standardized coding rules developed for the study (Available upon request). The final visit diagnosis was adjusted if the written diagnosis indicated an eligible infection presentation. Non-infection diagnoses (e.g.’new patient visit’) and non-eligible infections (eg. pyleonephritis, chronic sinusitis) were excluded, as were presentations involving asthma or chronic lung disease. Raters agreed on the final diagnosis for 94% of visits. Disagreements were resolved through case review and agreement by both raters.

Data abstracted from each visit included patient age, sex, antibiotic allergies, clinic site, visit date, clinician type, billing ICD-9 code, clinician written diagnosis, selected vital signs, tests ordered, and antibiotic prescriptions. Prescription information included the antibiotic name, prescription duration, and if a patient was advised to delay filling the prescription (‘delayed prescription’).

### Study outcomes

The primary study outcome was total antibiotic prescriptions for the five selected conditions combined (URI, sore throat presentations, acute sinusitis, acute bronchitis, acute uncomplicated cystitis) in each arm. Secondary outcomes were the proportion of prescriptions issued as delayed antibiotic prescriptions, prescriptions for longer than 7 days duration, and total, delayed and long duration prescriptions for each infection individually. A post-hoc decision was made to also assess test utilization.

### Statistical analysis

A 25% relative reduction in the total prescriptions was selected as the minimum important effect size, consistent with previously reported national goals [25]. To detect a 25% relative difference with 90% power, assuming a similar 30% antibiotic prescribing rate as in the pilot study [26], a sample size of 834 cases in each study arm was estimated, unadjusted for clustering. Visit and clinic characteristics of each group were compared using unadjusted chi-square, Fisher’s exact test or t-tests as appropriate. The intervention effects were expressed as odds ratios, estimated from unadjusted and adjusted logistic generalized estimation equation (GEE) models that accounted for clustering by clinics in each study arm. Models were adjusted for differing baseline characteristics between control and intervention groups associated with antibiotic prescriptions, as well as baseline prescribing rates. All analyses were performed using R statistical software [27].

## Results

**Fig. 1:**
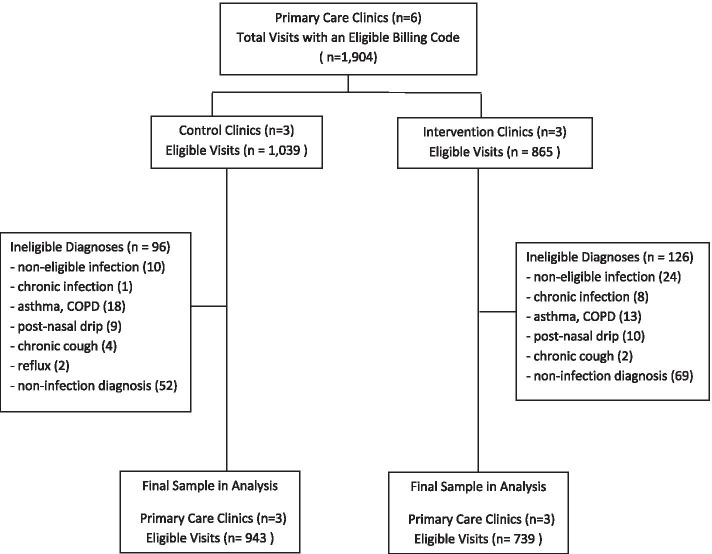
Clinic randomization, reasons for exclusions and final sample of eligible visits included in the analysis

There were 1,904 eligible visits from January 1, 2019 until May 31, 2019. After exclusions, 1682 (88.3%) visits were available for analysis; 943(56.1%) in control clinics and 739 (43.9%) from intervention clinics (Fig. 1). The average meeting attendance at intervention clinics was 81% with a 77% average education module completion rate. Overall, 16/34 (47.1%) of these providers completed all intervention elements.

Table 1 compares characteristics of intervention and control clinic visits in the prior winter and during the evaluation period. In both time periods, groups differed in the number of providers, total visits, and visits outside of regular clinic hours (after hours). Patients seen at intervention clinics during the evaluation tended to be older (mean 53.4 years) than at control clinics (47.1 years, p<0.001), more likely to be female (76.9% versus 70.6%, p=0.005), and have an antibiotic allergy (24.0% versus 16.1%, p<0.001). The distribution of infections differed between the groups in both time periods (p<0.001). While there were fewer cases of acute bronchitis at intervention clinics during the evaluation period (6.6%) compared to the baseline period (11.0%), there was no increase in pneumonia diagnoses to suggest diagnostic shifting (8.9% evaluation period vs 10.4% baseline).

**Table 1:** Comparison of control and intervention clinic characteristics during the baseline and evaluation periods

Characteristic	Baseline Period (January-February 2018)			Evaluation Period (January-May 2019)		
	Control	Intervention	p-value	Control	Intervention	p-value
Clinicians	19	31		20	33^d^	
Total visits^a^	634	328		943	739	
After hours visits	15 (2.4%)	47 (14.3%)	<0.001	36 (3.8%)	243 (32.9%)	<0.001
Visits involving residents	102 (16.1%)	56 (17.1%)	0.765	68 (7.2%)	123 (16.6%)	<0.001
Age (mean years, SD)	47.1 (16.8)	51.9 (17.6)	<0.001	47.1 (17.5)	53.4 (19.1)	<0.001
Visits by Females	446 (70.3%)	255 (77.7%)	0.018	666 (70.6%)	568 (76.9%)	0.005
Visits with an Antibiotic allergy History	111 (17.5%)	95 (29.0%)	<0.001	152 (16.1%)	177 (24.0%)	<0.001
Characteristic	Baseline Period (January-February 2018)			Intervention Period (January-May 2019)		
	Control	Intervention	p-value	Control	Intervention	p-value
Visits by Condition						
URI^b^	245 (38.6%)	92 (28.0%)		287 (30.4%)	185 (25.0%)	
Acute sinusitis	91 (14.4%)	69 (21.0%)		140 (14.8%)	133 (18.0%)	
Sore throat	60 (9.5%)	27 (8.2%)		91 (9.7%)	81 (11.0%)	
Acute bronchitis	67 (10.6%)	36 (11.0%)		126 (13.4%)	49 (6.6%)	
Acute cystitis	50 (7.9%)	43 (13.1%)		119 (12.6%)	161 (21.8%)	
Pneumonia	45 (7.1%)	34 (10.4%)		69 (7.3%)	66 (8.9%)	
Influenza	46 (7.3%)	22 (6.7%)		47 (5.0%)	15 (2.0%)	
Other^c^	30 (4.7%)	5 (1.5%)	<0.001	64 (6.8%)	49 (6.6%)	<0.001
Crude Prescribing Rate	218 (34.4%)	126 (38.4%)		373 (39.6%)	349 (47.2%)	

^a^ some patients may have had more than 1 visit ^b^URI –upper respiratory tract infection; ^c^ Other includes cough (ICD9 786), other urinary conditions (599); ^d^34 providers completed the education but 1 had no visit data

Factors associated with receiving an antibiotic prescription (Supplementary Table 1) included the type of infection (p<0.01,), being assessed after hours (p=0.02), female gender (p<0.01), and age (p=0.07). These factors were adjusted for in comparisons of prescribing outcomes. Table 2 indicates the intervention effects on prescribing outcomes for the five selected infections combined (n=1,372 visits). There was an observed increase in the crude odds of receiving any antibiotic prescription at intervention clinics (crude odds ratio (OR)=1.72; 95% CI =1.00, 2.98). However, after adjustment for characteristics associated with antibiotic prescriptions, there was a 22% reduction in the odds of receiving an antibiotic prescription (adjusted OR = 0.78; 95% CI = 0.64, 0.96) at intervention clinics.

**Table 2:** Comparison of antibiotic prescribing outcomes in intervention and control clinics for main infections combined^a^

Arm	N	Crude Rate	Unadjusted GEE Estimate		Adjusted GEE Estimate^c^	
			OR (95% CI^b^)	P	OR (95% CI)	p
Total Antibiotic Prescriptions						
Control	763	325 (42.6%)				
Intervention	609	297 (48.8%)	1.72 (1, 2.98)	0.05	0.78 (0.64, 0.96)	0.02
Delayed Antibiotic Prescriptions						
Control	324^d^	38 (11.7%)				
Intervention	294	65 (22.1%)	1.13 (0.47, 2.68)	0.79	2.29 (1.37, 3.83)	<0.01
Prescription Duration Longer than 7 Days^e^						
Control	273	80 (29.3%)				
Intervention	258	55 (21.3%)	0.34 (0.12, 1.01)	0.05	0.24 (0.13, 0.43)	<0.0001
First Line Antibiotic Choice^f^						
Control	232	197 (84.9%)				
Intervention	258	238 (92.2%)	2.2 (2.02, 2.4)	<0.0001	1.41 (0.66, 3.01)	0.37

^a^URI, sore throat, acute sinusitis, acute bronchitis, acute cystitis^b^ 95% confidence interval; ^c^adjusted for case-mix of conditions, age, sex, afterhours visits, and baseline prescribing rate; ^d^ denominators differ from total prescriptions in some cases due to missing data; ^e^ sore throat cases excluded from analysis ^f^excluding URI and bronchitis where any antibiotic is considered inappropriate

More prescriptions at intervention clinics were issued as delayed prescriptions (22.1%) than in control clinics (11.7%; adjusted OR = 2.29; 95% CI=1.37, 3.83). The odds that intervention physicians advised patients to start antibiotics immediately were reduced 37% compared to control clinics (adjusted OR= 0.63; 95% CI=0.45, 0.89). Antibiotic prescriptions for longer than 7 days duration were also decreased at interventions clinic (21.3%) compared to control clinics (29.3%; adjusted OR = 0.24; 95% CI = 0.13, 0.43). Use of recommended antibiotics were high in both control (84.9%) and intervention (92.2%) clinics (adjusted p=0.37).

There were no reductions in total antibiotic prescriptions for individual infections (Table 3). Prescriptions for URI cases were increased in intervention clinics (13.0% vs 7.7% control clinics, p<0.01), although most (56.5%) were delayed prescriptions. The odds of a delayed prescription was increased for acute sinusitis in unadjusted comparisons, and for acute cystitis in adjusted comparisons. The odds of a prescription for longer than 7 days was reduced for acute bronchitis only (adjusted OR = 0.46; 95% CI = 0.29, 0.73).

**Table 3:** Comparison of the main antibiotic prescribing outcomes in intervention and control clinics by condition

	Outcome	Control	Intervention	Unadjusted GEE Estimate		Adjusted GEE Estimate^a^	
				OR (95% CI^b^)	p	OR (95% CI)	p
URI	N	287	185				
	Total Antibiotic Prescriptions	22 (7.7%)	24 (13.0%)	1.68 (1.4, 2.02)	<0.0001	1.63 (1.2, 2.23)	<0.01
	Delayed Antibiotic Prescriptions	10 (45.5%)	13 (56.5%)	0.8 (0.13, 4.96)	0.81	0.94 (0.2, 4.46)	0.94
	Prescription Duration Longer than 7 days^c^	6 (27.3%)	6 (27.3%)	1.72 (1.04, 2.83)	0.03	1.32 (0.11, 16.5)	0.83
Sinusitis	N	140	133				
	Total Antibiotic Prescriptions	88 (62.9%)	94 (70.7%)	1.24 (1.04, 1.48)	0.02	1.45 (0.82, 2.57)	0.2
	Delayed Antibiotic Prescriptions	13 (14.8%)	23 (24.7%)	2.17 (1.3, 3.61)	<0.01	-^d^	
	Prescription Duration Longer than 7 days^c^	57 (64.8%)	43 (45.7%)	0.43 (0.21, 0.89)	0.02	-	
Sore Throat^c^	N	91	81				
	Total Antibiotic Prescriptions	52 (57.1%)	36 (44.4%)	1.0 (0.26, 3.89)	0.99	-^d^	
	Delayed Antibiotic Prescriptions	6 (11.5%)	6 (16.7%)	1.98 (0.37, 10.5)	0.42	-	
Bronchitis	N	126	49				
	Total Antibiotic Prescriptions	71 (56.3%)	15 (30.6%)	0.35 (0.27, 0.46)	<0.0001	0.52 (0.19, 1.39)	0.19
	Delayed Antibiotic Prescriptions	3 (4.3%)	4 (28.6%)	4.59 (0.45, 47.1)	0.2	8.48 (0.21, 338)	0.26
	Prescription Duration Longer than 7 days	13 (18.3%)	3 (20.0%)	0.69 (0.08, 5.98)	0.73	0.46 (0.29, 0.73)	<0.001
Cystitis	N	119	161				
	Total Antibiotic Prescriptions	92 (77.3%)	128 (79.5%)	1.12 (0.71, 1.79)	0.62	0.96 (0.6, 1.55)	0.87
	Delayed Antibiotic Prescriptions	6 (6.5%)	19 (14.8%)	3.68 (2.48, 5.47)	<0.0001	1.59 (1.08, 2.35)	0.02
	Prescription Duration Longer than 7 days3	4 (4.3%)	3 (2.4%)				

^a^adjusted for age, sex, afterhours visit, and baseline prescribing rate, except for cystitis which involved females only ^b^ 95% confidence interval; ^c^Sore throat cases excluded from duration analyses as 10 days recommended;^d^ Some analyses unable to be conducted due to too few cases during baseline period; ^e^Analyses excluded due to too few cases

A post-hoc analysis of the effect of testing recommendations included in clinical decision aids found a throat swab or rapid strep test was ordered more frequently at intervention clinics (56/81, 69.1%) than control clinics (28/91, 30.8%) for sore throat presentations (adjusted OR = 4.63, 95% CI=2.81, 7.63). Chest x-rays were ordered more often at intervention clinics (19/49, 38.8%) than control clinics (13/126, 10.3%) for cases of acute bronchitis (adjusted OR = 3.85, 95% CI = 2.58, 5.76). Urinalysis and urine culture utilization was similar in both control (93.3%) and intervention (90.7%) clinics.

## Discussion

This community-based, primary care provider-focused antimicrobial stewardship intervention reduced total antibiotic prescriptions to adults with a respiratory or urinary tract infection, increased the proportion issued as delayed prescriptions, and reduced prescription durations for longer than 7 days.

Although the observed crude rates of total antibiotic prescriptions were not different between intervention and control clinics, there were a number of differences in baseline characteristics between the two groups of clinics that persisted after randomization. When clinic difference were adjusted for, there was a 22% reduction in the odds of prescribing an antibiotic at intervention clinics. In addition, these prescriptions were twice as likely to be issued as delayed prescriptions. This is relevant as only 33% of prescriptions with instructions to delay starting may ultimately be filled [28]. As a result, patients seen at intervention clinics were less likely to be advised to begin antibiotics immediately. Prolonged antibiotic treatment durations are also an issue in primary care [29]. Long duration prescriptions were substantially reduced at intervention clinics. In addition, the change in test utilization at intervention clinics suggests the decision aids were important in affecting clinical change, as testing recommendations were only included in these aids.

Other multi-faceted ASP intervention studies have varied in the intervention focus, types of infections, and measures of antibiotic use [10, 30, 31]. A Quebec-based trial of shared decision making reported a 50% reduction in patient-reported intent to start antibiotics after a respiratory infection [30]. However, delayed prescriptions were allowed, which may have been later filled, and changes in total or dispensed antibiotics was not reported. A British trial of physician education, decision support and audit and feedback reported a 12% relative reduction in antibiotic prescriptions, but for respiratory infections only [31]. An evaluation of a national AMS program (TARGET) where practices were unaware they were part of a trial found a small 2.7% relative reduction in dispensed antibiotics [10]. These studies highlight the challenges of multi-faceted primary care AMS interventions, and the need to consider broader prescribing characteristics other than total prescriptions to fully understand the potential impact of primary care stewardship on antimicrobial resistance.

Providers were compensated for participating in the current study. An American study similarly provided compensation to participants with a 70% participation rate [32]. Incentives have also been implemented outside of studies to promote optimal antimicrobial use. In Britain, a financial Quality Premium introduced in 2015 for reductions in antibiotic use was associated with an 8% absolute decline in antibiotic prescriptions [33]. The current study also provided logistical support for AMS activities in intervention clinics. Other jurisdictions have similarly utilized direct supports to clinics [34, 35]. A Scottish national AMS program utilized Antimicrobial Management Teams to support local prescribing leadership [34]. In Britain, Medicine Management Teams play a role in education of community providers [35]. The current study and this research suggest the provision of incentives and logistical support at the clinic level may be important in facilitating community AMS efforts.

A limitation of the study was not all clinicians at each clinic participated. Participating clinicians may have been more receptive to the AMS intervention than might be seen in the broader primary care community. Another limitation was the short study duration over one winter season. Whether the impact on prescriptions would be sustained is unclear. AMS effects have declined when interventions are removed [36, 37]. The study also relied on written prescriptions as an outcome rather than dispensed antibiotics. While reducing initial prescriptions and their durations are important to reducing dispensed antibiotics, patients may have sought out other providers and received antibiotics. However, as more providers are exposed to AMS practices, patients may receive consistent antibiotic recommendations from all prescribers. Finally, the safety of this program was not assessed. Other studies have found adverse events associated with reduced antibiotic use in primary care are uncommon and require large samples to detect [38]. However, the elderly may be one group where caution is needed [39]. Clear ‘safety-netting advice’ where antibiotics are not prescribed, appropriate use of delayed prescriptions and clinical follow up can help mitigate such risks and prevent a loss of provider confidence in stewardship efforts.

## Conclusion

This study has demonstrated that clinically important changes in antibiotic utilization in primary care clinics are possible with local stewardship efforts involving provider-focused education, clinical decision aids, clinic support, ongoing audit and feedback, and compensation for ASP activities. Structured and supported community-based antimicrobial stewardship efforts, similar to those in hospital settings, warrant further study.

## Supplementary Information

**Additional file 1: Supplementary Table 1**. Associations between visit, patient, and condition characteristics, and having received an antibiotic prescription.

## Data Availability

The data is available by contacting the principal author on reasonable request. Appropriate data transfer agreements with the participating institutions may be required.
