# Supplementary material for: A pragmatic randomized trial of a primary care antimicrobial stewardship intervention in Ontario, Canada
Source: BMC Fam Pract. 2021 Sep 15;22:185. doi: 10.1186/s12875-021-01536-3 (PMC8442308; doi:10.1186/s12875-021-01536-3)
Supplement: Supplementary file 1 — Additional file 1: Supplementary Table 1. Associations between visit, patient, and condition characteristics, and having received an antibiotic prescription. [file 12875_2021_1536_MOESM1_ESM.docx]

Supplementary Table 1 – Associations between visit, patient, and condition characteristics, and having received an antibiotic prescription

| Characteristic | No Antibiotic  (n=960) | Antibiotic  (n=722) | *p* |
| --- | --- | --- | --- |
| *Antibiotic Prescriptions by Visit Type*  Any Visit  Provider Type^2^   - Staff Physician - Nurse Practitioner   After Hour Visits   - Yes - No   Visits with a Resident   - Yes - No | N (%)  960 (57.1%)  959 (57.2%)  1 (50.0%)  141 (50.5%)  819 (58.4%)  128 (55.8%)  832 (67.0%) | N (%)^1^  722 (42.9%)  719 (42.9%)  1 (50.0%)  138 (49.5%)  584 (41.6%)  63 (44.2%)  659 (33.0%) | -  0.49  0.02  <0.01 |
| *Antibiotic Prescriptions by Patient Characteristics*  Age, mean (SD)  Sex   - Female - Male | 49.1 (18.4)  668 (54.1%)  292 (65.2%) | 50.8 (18.5)  566 (45.9%)  156 (34.8%) | 0.07  <0.01 |

Supplementary Table 1 – Associations between visit, patient, and condition characteristics, and having received an antibiotic prescription (continued).

| Characteristic | No Antibiotic  (n=960) | Antibiotic  (n=722) | *p* |
| --- | --- | --- | --- |
| *Antibiotic Prescriptions by Patient Characteristics*  Antibiotic allergy history   - Yes - No   *Condition*  URI visits  Sinusitis visits  Sore Throat visits  Bronchitis visits  Cystitis visits  Other^3^ condition visits | 176 (53.5%)  784 (57.9%)  426 (90.3%)  91 (33.3%)  84 (48.8%)  89 (50.9%  60 (21.4%)  210 (67.7%) | 153 (46.5%)  569 (42.1%)  46 (9.7%)  182 (66.7%)  88 (51.2%)  86 (49.1%)  220 (78.6%)  100 (32.3%) | 0.2  <0.01 |

^1^ percentages are row percentages; ^2.^ Total cases may differ for some variables due to missing data;  ^3^ Other - includes cough (ICD9 786), other urinary conditions (599), pneumonia (486), influenza (487)
